# Supplementary material for: Individual and combined effect of organic eutrophication (DOC) and ocean warming on the ecophysiology of the Octocoral Pinnigorgia flava
Source: PeerJ. 2023 Feb 17;11:e14812. doi: 10.7717/peerj.14812 (PMC9940650; doi:10.7717/peerj.14812)
Supplement: Supplemental Information 1 — The raw data shows the ecophysiological response of the gorgonian Pinnigorgia flava to the effect of organic enrichment and increased water temperatures. In particular, the files report the measurements of oxygen concentration and the coral surfaces under the individual and the combined effect of DOC and temperatures throughout the whole experiment duration. [file peerj-11-14812-s001.zip › DOCTE - New - Raw Data/DOCTE - Readme - Raw data.rtf]

Readme Data files clarification for columns interpretation:day - time point recorded according to experimental days since the start of the experimental manipulations. (-1) Represents data collected as base line, one day before the start of treatment manipulations. treatment - column representing the DOC levels into the experiment, which were:         control        (2-3 mg/l = control) 		10              (10 mg/l = Low)   	20	          (20 mg/l = Medium)	40	          (40 mg/l = High)	controlT     (2-3 mg/l = Control) - Constant temperature control for experimental phase two.temperature - column representing the temperature levels into the experiment, which were: 	26 ºC, 28 ºC, 30 ºC, 32 ºC resp - measured values for respiration, i.e., Oxygen consumption.phot - measured values for net photosynthesis, i.e., Oxygen production.symbiodiniaceae_density - measured symbiont density.change_in_surface_area - calculated changes in the P. flava coral fragments surface area in our experiments.    id_aquaria - numeric variable representing aquaria id.                   	- for the initial stage of the experiment: 12 aquaria		 	- for the second stage of the experiment: 16 aquariaid_colony - dummy variable depicting a combination of a number and a letter where, the number represents the individual coral fragment identifier (a number between 1 and 10), and the letter represents the aquaria to which the coral fragmentbelongs to: A to L for aquaria from 1 to 12 in the first phase of experiments, and A to P for aquaria from 1 to 16 in the second phase of experiments. Additional Note: the 10 coral fragments in each experimental aquaria were propagated from three clonal mother colonies keptin our aquaria facilities. These colonies were randomly harvested and subsequently randomly allocated in each of the experimental tanks. Once allocated, each colony in each tank received an identifiable ID marking from 1 to 10 in order to keep records of each of the individuals  during our experimental procedures. From the available coral fragments in each tank, a total of 160 P. flava fragments were used for this particular study.
