# Supplementary material for: Individual and combined effect of organic eutrophication (DOC) and ocean warming on the ecophysiology of the Octocoral Pinnigorgia flava
Source: PeerJ. 2023 Feb 17;11:e14812. doi: 10.7717/peerj.14812 (PMC9940650; doi:10.7717/peerj.14812)
Supplement: Supplemental Information 1 — The raw data shows the ecophysiological response of the gorgonian Pinnigorgia flava to the effect of organic enrichment and increased water temperatures. In particular, the files report the measurements of oxygen concentration and the coral surfaces under the individual and the combined effect of DOC and temperatures throughout the whole experiment duration. [file peerj-11-14812-s001.zip › DOCTE - New Supplementary Materials/New - Supplementary Materials.docx]

**Supplementary Materials**

**S1. Controlled experimental background parameters measured through the study.**

**S1.1.** Summary of background parameters measured through the complete experiment depicting mean values ± SD for every tank across all treatments.

Note: the values presented here for flow rates (L s^-1^) and turnover time (s) were obtained via calculating their corresponding theoretical values for each tank according to Grottoli et.al. (2021). Here, flow rate was calculated in each tank as a volumetric water flow rate per unit time, which in a closed system tank would correspond to the fluid output from the exhaust of the pump. On the other hand, the water turnover time would correspond to the time required to replace the entire volume of water in the tank, assuming the tank is continuously well mixed. This values was calculated here for each tank, by dividing the tank volume by it corresponding flow rate.

**S1.2.** Summary of background parameters prior to the start of the first experimental stage of the study corresponding to individual DOC additions. The table shows mean values ± SD for every tank across all treatments.

**S1.3.** Summary of background parameters prior to the start of the second experimental stage of the study, right after individual DOC addition and before implementing increased temperature treatments. The table shows mean values ± SD for every tank across all treatments.

**S2. Statistical background parameters comparisons**

**S2.1.** Statistical results for background parameters comparison through the complete experiment. The statistical analysis was performed via a simple LM where contrasts were done across all experimental treatments including: the DOC control tanks (Control), the temperature control tanks (ControlT), the initial maintenance tank where the colonies were breed, and the tanks corresponding to the DOC manipulated conditions 10, 20 and 40 mg/l. There were no significant differences between tanks regarding the background parameters presented in the table, and all tanks showed comparable conditions except for waterflow related parameters in the maintenance tank. These water flow parameters were not included here but were significantly different (LM; p < 0.05) as expected according to the values observed in supplementary materials S1.

| **Parameter** | ***df*** | **F** | ***p*** |
| --- | --- | --- | --- |
| **Light** (µmol m^−2^ s^−1^) | 5 | 0.8202 | 0.5601 |
| **Salinity** (ppt) | 5 | 0.2922 | 0.9074 |
| **pH** | 5 | 2.0622 | 0.1473 |
| **KH** | 5 | 2.3253 | 0.1131 |
| **Ca** (ppm) | 5 | 1.4526 | 0.2808 |
| **Mg** (ppm) | 5 | 1.2441 | 0.3529 |

**S2.2.** Statistical results for background parameters prior to the start of the first and second experimental stages, respectively. The parameters were statistically analysed via simple LM contrasting across experimental treatments. For the analysis corresponding to the parameters prior to the start of the first stage, we compared: the DOC control tanks (Control), the initial maintenance tank where colonies were breed, and the corresponding tanks to the DOC manipulated conditions 10, 20 and 40 mg/l. In addition, for the analysis corresponding to the second experimental stage, we included the temperature control tanks (ControlT) and compared the DOC parameter only among the tanks representing control conditions and the maintenance tank. No significant differences were found in any of the background parameters shown in the table, except for the waterflow related parameters in the maintenance tank (not included here, LM; p < 0.05; please see supplementary materials S1 for further reference on raw data values).

|  | **Stage 1** | | | **Stage 2** | | |
| --- | --- | --- | --- | --- | --- | --- |
| **Parameter** | ***df*** | **F** | ***p*** | ***df*** | **F** | ***p*** |
| **Light** (µmol m^−2^ s^−1^) | 4 | 0.9605 | 0.4786 | 5 | 0.5784 | 0.7162 |
| **Temperature** (°C) | 4 | 2.4185 | 0.1339 | 5 | 1.3067 | 0.3294 |
| **DOC** (mg/l) | 4 | 0.4064 | 0.7994 | 2 | 0.5932 | 0.5873 |
| **Salinity** (ppt) | 4 | 2.4697 | 0.1287 | 5 | 1.8451 | 0.1845 |
| **pH** | 4 | 3.0769 | 0.08247 | 5 | 0.5405 | 0.7424 |
| **KH** | 4 | 1.4615 | 0.2997 | 5 | 0.5176 | 0.7582 |
| **Ca** (ppm) | 4 | 1.4327 | 0.3076 | 5 | 2.7276 | 0.07687 |
| **Mg** (ppm) | 4 | 0.9982 | 0.4617 | 5 | 0.1475 | 0.9766 |

**S3. Post hoc model analyses: Tukey test results summary tables**

**S3.1.** Post-hoc multiple comparisons test results for *P. flava* O_2_ production assessment during the experiments' second stage, including warming and DOC addition treatments.

| **Contrast** | ***df*** | **p** |
| --- | --- | --- |
| 26-28 | 44.6 | 0.9995 |
| 26-30 | 44.0 | 0.3003 |
| 26-32 | 44.0 | **0.0024 **** |
| 28-30 | 44.6 | 0.2640 |
| 28-32 | 44.6 | **0.0021 **** |
| 30-32 | 44.0 | 0.1939 |

**S3.2.** Post-hoc comparison test results for *P. flava* fragments change in surface area: simplified initial assessment of the significant interaction term between temperature and DOC, fixing the DOC treatment factor intercept and varying it across temperatures.

|  | **Estimates for Temperature** | | | **Contrast by DOC Treatment** | |
| --- | --- | --- | --- | --- | --- |
| **Fixed Factors** | **28 ºC** | **30 ºC** | **32 ºC** | ***df*** | **p** |
| ControlT | 0.0305 | -0.0826 | 0.4408 | 3 | **0.0091 **** |
| Control | 0.2468 | 0.2041 | 0.4400 | 3 | 0.0875 |
| High | 0.1398 | 0.1338 | -0.1128 | 3 | 0.2877 |
| Low | -0.3875 | -0.3573 | -0.4305 | 3 | **0.0046 **** |
| Medium | -0.1600 | -0.0021 | -0.2609 | 3 | 0.1071 |

**S3.3.** Model marginal means for the *P. flava* fragments change in surface area depicting the contrasts across DOC treatments and at each temperature treatment.

**S3.4.** Detailed results of Post-hoc pair comparisons test for *P. flava* fragments change in surface area. Complete summary of the significant interaction term assessment between temperature and DOC treatment pairs. Please see previous figure S3.3 for graphical reference of these results. Here, ControlT represents the control that remained always at 26 degrees during the second phase experiments while the increased temperature treatments correspond to the DOC control tanks (Control) and the DOC manipulated conditions 10 (Low), 20 (Medium) and 40 (High) mg/l. The contrasts are shown in the table as “DOC treatment.temperature - DOC treatment.temperature”. With this notation we indicate pair comparisons performed per each DOC condition at each increased temperature point. Here, e.g., “Low.30 – High.32” would represent the comparison between Low DOC treatment when temperature had been increased at 30 C contrasted to High DOC treatment when the temperature had been increased at 32 C. For the special case of ControlT, e.g., “ControlT.28 – Medium.32” would represent the comparison between ControlT at 26 C temperature, but at the time point where the rest of the experimental tanks were exposed to 28 C; contrasted to the Medium DOC treatment when the tanks were exposed to 32 C, and so on. Significant differences are highlighted in bold.

| **contrast** | **estimate** | **SE** | ***df*** | **t.ratio** | **p** |
| --- | --- | --- | --- | --- | --- |
| ControlT.26 - Control.26 | -0.4021 | 0.1488 | 30.1314 | -2.7030 | 0.4607 |
| ControlT.26 - Low.26 | -0.1267 | 0.1481 | 33.8046 | -0.8554 | 1.0000 |
| ControlT.26 - Medium.26 | -0.1493 | 0.1481 | 33.8046 | -1.0080 | 0.9999 |
| ControlT.26 - High.26 | -0.3120 | 0.1481 | 33.8046 | -2.1065 | 0.8317 |
| ControlT.26 - ControlT.28 | 0.1131 | 0.0931 | 3.7525 | 1.2146 | 0.9887 |
| ControlT.26 - Control.28 | -0.3594 | 0.1413 | 29.7706 | -2.5427 | 0.5643 |
| ControlT.26 - Low.28 | -0.1569 | 0.1481 | 33.8046 | -1.0592 | 0.9999 |
| ControlT.26 - Medium.28 | -0.3071 | 0.1481 | 33.8046 | -2.0737 | 0.8478 |
| ControlT.26 - High.28 | -0.3060 | 0.1555 | 43.8959 | -1.9683 | 0.8979 |
| ControlT.26 - ControlT.30 | -0.4103 | 0.1425 | 43.3399 | -2.8786 | 0.3427 |
| **ControlT.26 - Control.30** | **-0.5954** | **0.1488** | **30.1314** | **-4.0022** | **0.0367 *** |
| ControlT.26 - Low.30 | -0.0837 | 0.1481 | 33.8046 | -0.5651 | 1.0000 |
| ControlT.26 - Medium.30 | -0.0484 | 0.1481 | 33.8046 | -0.3267 | 1.0000 |
| ControlT.26 - High.30 | -0.0594 | 0.1555 | 43.8959 | -0.3818 | 1.0000 |
| ControlT.26 - ControlT.32 | 0.0305 | 0.1168 | 5.6304 | 0.2610 | 1.0000 |
| ControlT.26 - Control.32 | -0.1553 | 0.1555 | 43.8959 | -0.9990 | 1.0000 |
| ControlT.26 - Low.32 | -0.5142 | 0.1481 | 33.8046 | -3.4715 | 0.1143 |
| ControlT.26 - Medium.32 | -0.3093 | 0.1481 | 33.8046 | -2.0881 | 0.8408 |
| ControlT.26 - High.32 | -0.1722 | 0.1481 | 33.8046 | -1.1625 | 0.9996 |
| Control.26 - Low.26 | 0.2754 | 0.1629 | 35.2032 | 1.6905 | 0.9697 |
| Control.26 - Medium.26 | 0.2528 | 0.1629 | 35.2032 | 1.5517 | 0.9868 |
| Control.26 - High.26 | 0.0901 | 0.1629 | 35.2032 | 0.5530 | 1.0000 |
| Control.26 - ControlT.28 | 0.5151 | 0.1488 | 30.1314 | 3.4631 | 0.1223 |
| Control.26 - Control.28 | 0.0427 | 0.0915 | 3.2398 | 0.4663 | 1.0000 |
| Control.26 - Low.28 | 0.2452 | 0.1629 | 35.2032 | 1.5052 | 0.9904 |
| Control.26 - Medium.28 | 0.0950 | 0.1629 | 35.2032 | 0.5829 | 1.0000 |
| Control.26 - High.28 | 0.0961 | 0.1696 | 43.7439 | 0.5665 | 1.0000 |
| Control.26 - ControlT.30 | -0.0082 | 0.1579 | 43.1130 | -0.0521 | 1.0000 |
| Control.26 - Control.30 | -0.1933 | 0.1505 | 6.5945 | -1.2842 | 0.9913 |
| Control.26 - Low.30 | 0.3184 | 0.1629 | 35.2032 | 1.9544 | 0.8995 |
| Control.26 - Medium.30 | 0.3537 | 0.1629 | 35.2032 | 2.1712 | 0.7983 |
| Control.26 - High.30 | 0.3427 | 0.1696 | 43.7439 | 2.0206 | 0.8765 |
| Control.26 - ControlT.32 | 0.4326 | 0.1515 | 30.5134 | 2.8558 | 0.3688 |
| Control.26 - Control.32 | 0.2468 | 0.1696 | 43.7439 | 1.4549 | 0.9940 |
| Control.26 - Low.32 | -0.1121 | 0.1629 | 35.2032 | -0.6880 | 1.0000 |
| Control.26 - Medium.32 | 0.0928 | 0.1629 | 35.2032 | 0.5698 | 1.0000 |
| Control.26 - High.32 | 0.2299 | 0.1629 | 35.2032 | 1.4113 | 0.9953 |
| Low.26 - Medium.26 | -0.0226 | 0.1623 | 38.0337 | -0.1393 | 1.0000 |
| Low.26 - High.26 | -0.1853 | 0.1623 | 38.0337 | -1.1416 | 0.9997 |
| Low.26 - ControlT.28 | 0.2397 | 0.1481 | 33.8046 | 1.6187 | 0.9796 |
| Low.26 - Control.28 | -0.2327 | 0.1562 | 35.1747 | -1.4901 | 0.9914 |
| Low.26 - Low.28 | -0.0302 | 0.1279 | 4.9981 | -0.2362 | 1.0000 |
| Low.26 - Medium.28 | -0.1804 | 0.1623 | 38.0337 | -1.1117 | 0.9998 |
| Low.26 - High.28 | -0.1793 | 0.1691 | 43.9963 | -1.0607 | 0.9999 |
| Low.26 - ControlT.30 | -0.2836 | 0.1573 | 43.9196 | -1.8036 | 0.9494 |
| Low.26 - Control.30 | -0.4687 | 0.1629 | 35.2032 | -2.8768 | 0.3512 |
| Low.26 - Low.30 | 0.0430 | 0.1623 | 38.0337 | 0.2648 | 1.0000 |
| Low.26 - Medium.30 | 0.0783 | 0.1623 | 38.0337 | 0.4824 | 1.0000 |
| Low.26 - High.30 | 0.0673 | 0.1691 | 43.9963 | 0.3983 | 1.0000 |
| Low.26 - ControlT.32 | 0.1572 | 0.1508 | 34.1036 | 1.0420 | 0.9999 |
| Low.26 - Control.32 | -0.0286 | 0.1691 | 43.9963 | -0.1693 | 1.0000 |
| Low.26 - Low.32 | -0.3875 | 0.1623 | 38.0337 | -2.3871 | 0.6667 |
| Low.26 - Medium.32 | -0.1826 | 0.1623 | 38.0337 | -1.1248 | 0.9997 |
| Low.26 - High.32 | -0.0455 | 0.1623 | 38.0337 | -0.2803 | 1.0000 |
| Medium.26 - High.26 | -0.1627 | 0.1623 | 38.0337 | -1.0023 | 0.9999 |
| Medium.26 - ControlT.28 | 0.2624 | 0.1481 | 33.8046 | 1.7714 | 0.9535 |
| Medium.26 - Control.28 | -0.2101 | 0.1562 | 35.1747 | -1.3453 | 0.9973 |
| Medium.26 - Low.28 | -0.0076 | 0.1623 | 38.0337 | -0.0467 | 1.0000 |
| Medium.26 - Medium.28 | -0.1578 | 0.1279 | 4.9981 | -1.2345 | 0.9912 |
| Medium.26 - High.28 | -0.1567 | 0.1691 | 43.9963 | -0.9269 | 1.0000 |
| Medium.26 - ControlT.30 | -0.2610 | 0.1573 | 43.9196 | -1.6598 | 0.9764 |
| Medium.26 - Control.30 | -0.4461 | 0.1629 | 35.2032 | -2.7380 | 0.4344 |
| Medium.26 - Low.30 | 0.0656 | 0.1623 | 38.0337 | 0.4041 | 1.0000 |
| Medium.26 - Medium.30 | 0.1009 | 0.1623 | 38.0337 | 0.6217 | 1.0000 |
| Medium.26 - High.30 | 0.0899 | 0.1691 | 43.9963 | 0.5321 | 1.0000 |
| Medium.26 - ControlT.32 | 0.1798 | 0.1508 | 34.1036 | 1.1919 | 0.9994 |
| Medium.26 - Control.32 | -0.0060 | 0.1691 | 43.9963 | -0.0355 | 1.0000 |
| Medium.26 - Low.32 | -0.3649 | 0.1623 | 38.0337 | -2.2478 | 0.7552 |
| Medium.26 - Medium.32 | -0.1600 | 0.1623 | 38.0337 | -0.9855 | 1.0000 |
| Medium.26 - High.32 | -0.0229 | 0.1623 | 38.0337 | -0.1410 | 1.0000 |
| High.26 - ControlT.28 | 0.4251 | 0.1481 | 33.8046 | 2.8698 | 0.3568 |
| High.26 - Control.28 | -0.0474 | 0.1562 | 35.1747 | -0.3036 | 1.0000 |
| High.26 - Low.28 | 0.1551 | 0.1623 | 38.0337 | 0.9556 | 1.0000 |
| High.26 - Medium.28 | 0.0049 | 0.1623 | 38.0337 | 0.0300 | 1.0000 |
| High.26 - High.28 | 0.0060 | 0.1691 | 43.9963 | 0.0354 | 1.0000 |
| High.26 - ControlT.30 | -0.0983 | 0.1573 | 43.9196 | -0.6252 | 1.0000 |
| High.26 - Control.30 | -0.2834 | 0.1629 | 35.2032 | -1.7394 | 0.9608 |
| High.26 - Low.30 | 0.2283 | 0.1623 | 38.0337 | 1.4064 | 0.9956 |
| High.26 - Medium.30 | 0.2636 | 0.1623 | 38.0337 | 1.6240 | 0.9799 |
| High.26 - High.30 | 0.2526 | 0.1691 | 43.9963 | 1.4944 | 0.9920 |
| High.26 - ControlT.32 | 0.3425 | 0.1508 | 34.1036 | 2.2705 | 0.7402 |
| High.26 - Control.32 | 0.1567 | 0.1691 | 43.9963 | 0.9268 | 1.0000 |
| High.26 - Low.32 | -0.2022 | 0.1623 | 38.0337 | -1.2455 | 0.9990 |
| High.26 - Medium.32 | 0.0027 | 0.1623 | 38.0337 | 0.0168 | 1.0000 |
| High.26 - High.32 | 0.1398 | 0.1279 | 4.9981 | 1.0936 | 0.9970 |
| ControlT.28 - Control.28 | -0.4725 | 0.1413 | 29.7706 | -3.3425 | 0.1568 |
| ControlT.28 - Low.28 | -0.2699 | 0.1481 | 33.8046 | -1.8226 | 0.9410 |
| ControlT.28 - Medium.28 | -0.4202 | 0.1481 | 33.8046 | -2.8370 | 0.3756 |
| ControlT.28 - High.28 | -0.4191 | 0.1555 | 43.8959 | -2.6955 | 0.4564 |
| ControlT.28 - ControlT.30 | -0.5234 | 0.1425 | 43.3399 | -3.6717 | 0.0639 |
| **ControlT.28 - Control.30** | **-0.7084** | **0.1488** | **30.1314** | **-4.7623** | **0.0055 **** |
| ControlT.28 - Low.30 | -0.1968 | 0.1481 | 33.8046 | -1.3284 | 0.9976 |
| ControlT.28 - Medium.30 | -0.1614 | 0.1481 | 33.8046 | -1.0900 | 0.9998 |
| ControlT.28 - High.30 | -0.1724 | 0.1555 | 43.8959 | -1.1090 | 0.9998 |
| ControlT.28 - ControlT.32 | -0.0826 | 0.1168 | 5.6304 | -0.7070 | 1.0000 |
| ControlT.28 - Control.32 | -0.2684 | 0.1555 | 43.8959 | -1.7262 | 0.9658 |
| **ControlT.28 - Low.32** | **-0.6272** | **0.1481** | **33.8046** | **-4.2348** | **0.0184 *** |
| ControlT.28 - Medium.32 | -0.4223 | 0.1481 | 33.8046 | -2.8514 | 0.3673 |
| ControlT.28 - High.32 | -0.2852 | 0.1481 | 33.8046 | -1.9259 | 0.9092 |
| Control.28 - Low.28 | 0.2025 | 0.1562 | 35.1747 | 1.2967 | 0.9983 |
| Control.28 - Medium.28 | 0.0523 | 0.1562 | 35.1747 | 0.3347 | 1.0000 |
| Control.28 - High.28 | 0.0534 | 0.1632 | 43.8539 | 0.3273 | 1.0000 |
| Control.28 - ControlT.30 | -0.0509 | 0.1509 | 43.3229 | -0.3374 | 1.0000 |
| Control.28 - Control.30 | -0.2359 | 0.1249 | 5.0981 | -1.8897 | 0.8624 |
| Control.28 - Low.30 | 0.2757 | 0.1562 | 35.1747 | 1.7653 | 0.9554 |
| Control.28 - Medium.30 | 0.3110 | 0.1562 | 35.1747 | 1.9915 | 0.8849 |
| Control.28 - High.30 | 0.3001 | 0.1632 | 43.8539 | 1.8389 | 0.9403 |
| Control.28 - ControlT.32 | 0.3899 | 0.1442 | 30.1838 | 2.7037 | 0.4602 |
| Control.28 - Control.32 | 0.2041 | 0.1632 | 43.8539 | 1.2509 | 0.9990 |
| Control.28 - Low.32 | -0.1548 | 0.1562 | 35.1747 | -0.9909 | 1.0000 |
| Control.28 - Medium.32 | 0.0501 | 0.1562 | 35.1747 | 0.3211 | 1.0000 |
| Control.28 - High.32 | 0.1872 | 0.1562 | 35.1747 | 1.1988 | 0.9994 |
| Low.28 - Medium.28 | -0.1502 | 0.1623 | 38.0337 | -0.9256 | 1.0000 |
| Low.28 - High.28 | -0.1491 | 0.1691 | 43.9963 | -0.8821 | 1.0000 |
| Low.28 - ControlT.30 | -0.2534 | 0.1573 | 43.9196 | -1.6116 | 0.9823 |
| Low.28 - Control.30 | -0.4385 | 0.1629 | 35.2032 | -2.6915 | 0.4640 |
| Low.28 - Low.30 | 0.0732 | 0.1623 | 38.0337 | 0.4509 | 1.0000 |
| Low.28 - Medium.30 | 0.1085 | 0.1623 | 38.0337 | 0.6684 | 1.0000 |
| Low.28 - High.30 | 0.0975 | 0.1691 | 43.9963 | 0.5769 | 1.0000 |
| Low.28 - ControlT.32 | 0.1874 | 0.1508 | 34.1036 | 1.2422 | 0.9990 |
| Low.28 - Control.32 | 0.0016 | 0.1691 | 43.9963 | 0.0093 | 1.0000 |
| Low.28 - Low.32 | -0.3573 | 0.1623 | 38.0337 | -2.2011 | 0.7827 |
| Low.28 - Medium.32 | -0.1524 | 0.1623 | 38.0337 | -0.9388 | 1.0000 |
| Low.28 - High.32 | -0.0153 | 0.1623 | 38.0337 | -0.0943 | 1.0000 |
| Medium.28 - High.28 | 0.0011 | 0.1691 | 43.9963 | 0.0067 | 1.0000 |
| Medium.28 - ControlT.30 | -0.1032 | 0.1573 | 43.9196 | -0.6561 | 1.0000 |
| Medium.28 - Control.30 | -0.2882 | 0.1629 | 35.2032 | -1.7692 | 0.9546 |
| Medium.28 - Low.30 | 0.2234 | 0.1623 | 38.0337 | 1.3765 | 0.9966 |
| Medium.28 - Medium.30 | 0.2588 | 0.1623 | 38.0337 | 1.5941 | 0.9833 |
| Medium.28 - High.30 | 0.2478 | 0.1691 | 43.9963 | 1.4657 | 0.9936 |
| Medium.28 - ControlT.32 | 0.3376 | 0.1508 | 34.1036 | 2.2383 | 0.7595 |
| Medium.28 - Control.32 | 0.1518 | 0.1691 | 43.9963 | 0.8981 | 1.0000 |
| Medium.28 - Low.32 | -0.2070 | 0.1623 | 38.0337 | -1.2754 | 0.9987 |
| Medium.28 - Medium.32 | -0.0021 | 0.1623 | 38.0337 | -0.0131 | 1.0000 |
| Medium.28 - High.32 | 0.1349 | 0.1623 | 38.0337 | 0.8314 | 1.0000 |
| High.28 - ControlT.30 | -0.1043 | 0.1642 | 39.7100 | -0.6353 | 1.0000 |
| High.28 - Control.30 | -0.2893 | 0.1696 | 43.7439 | -1.7058 | 0.9693 |
| High.28 - Low.30 | 0.2223 | 0.1691 | 43.9963 | 1.3150 | 0.9982 |
| High.28 - Medium.30 | 0.2576 | 0.1691 | 43.9963 | 1.5239 | 0.9901 |
| High.28 - High.30 | 0.2467 | 0.1755 | 39.7100 | 1.4051 | 0.9958 |
| High.28 - ControlT.32 | 0.3365 | 0.1581 | 43.8936 | 2.1288 | 0.8246 |
| High.28 - Control.32 | 0.1507 | 0.1755 | 39.7100 | 0.8585 | 1.0000 |
| High.28 - Low.32 | -0.2082 | 0.1691 | 43.9963 | -1.2313 | 0.9992 |
| High.28 - Medium.32 | -0.0033 | 0.1691 | 43.9963 | -0.0193 | 1.0000 |
| High.28 - High.32 | 0.1338 | 0.1691 | 43.9963 | 0.7916 | 1.0000 |
| ControlT.30 - Control.30 | -0.1850 | 0.1579 | 43.1130 | -1.1722 | 0.9996 |
| ControlT.30 - Low.30 | 0.3266 | 0.1573 | 43.9196 | 2.0770 | 0.8507 |
| ControlT.30 - Medium.30 | 0.3619 | 0.1573 | 43.9196 | 2.3016 | 0.7233 |
| ControlT.30 - High.30 | 0.3510 | 0.1642 | 39.7100 | 2.1374 | 0.8185 |
| ControlT.30 - ControlT.32 | 0.4408 | 0.1454 | 43.3512 | 3.0322 | 0.2607 |
| ControlT.30 - Control.32 | 0.2550 | 0.1642 | 39.7100 | 1.5530 | 0.9874 |
| ControlT.30 - Low.32 | -0.1039 | 0.1573 | 43.9196 | -0.6604 | 1.0000 |
| ControlT.30 - Medium.32 | 0.1010 | 0.1573 | 43.9196 | 0.6426 | 1.0000 |
| ControlT.30 - High.32 | 0.2381 | 0.1573 | 43.9196 | 1.5143 | 0.9908 |
| Control.30 - Low.30 | 0.5117 | 0.1629 | 35.2032 | 3.1407 | 0.2209 |
| Control.30 - Medium.30 | 0.5470 | 0.1629 | 35.2032 | 3.3575 | 0.1433 |
| Control.30 - High.30 | 0.5360 | 0.1696 | 43.7439 | 3.1600 | 0.2029 |
| **Control.30 - ControlT.32** | **0.6258** | **0.1515** | **30.5134** | **4.1317** | **0.0265 *** |
| Control.30 - Control.32 | 0.4400 | 0.1696 | 43.7439 | 2.5943 | 0.5248 |
| Control.30 - Low.32 | 0.0812 | 0.1629 | 35.2032 | 0.4984 | 1.0000 |
| Control.30 - Medium.32 | 0.2861 | 0.1629 | 35.2032 | 1.7561 | 0.9574 |
| Control.30 - High.32 | 0.4232 | 0.1629 | 35.2032 | 2.5976 | 0.5257 |
| Low.30 - Medium.30 | 0.0353 | 0.1623 | 38.0337 | 0.2176 | 1.0000 |
| Low.30 - High.30 | 0.0243 | 0.1691 | 43.9963 | 0.1440 | 1.0000 |
| Low.30 - ControlT.32 | 0.1142 | 0.1508 | 34.1036 | 0.7570 | 1.0000 |
| Low.30 - Control.32 | -0.0716 | 0.1691 | 43.9963 | -0.4235 | 1.0000 |
| Low.30 - Low.32 | -0.4305 | 0.1279 | 4.9981 | -3.3670 | 0.3246 |
| Low.30 - Medium.32 | -0.2256 | 0.1623 | 38.0337 | -1.3896 | 0.9962 |
| Low.30 - High.32 | -0.0885 | 0.1623 | 38.0337 | -0.5451 | 1.0000 |
| Medium.30 - High.30 | -0.0110 | 0.1691 | 43.9963 | -0.0649 | 1.0000 |
| Medium.30 - ControlT.32 | 0.0789 | 0.1508 | 34.1036 | 0.5228 | 1.0000 |
| Medium.30 - Control.32 | -0.1069 | 0.1691 | 43.9963 | -0.6325 | 1.0000 |
| Medium.30 - Low.32 | -0.4658 | 0.1623 | 38.0337 | -2.8695 | 0.3525 |
| Medium.30 - Medium.32 | -0.2609 | 0.1279 | 4.9981 | -2.0405 | 0.8071 |
| Medium.30 - High.32 | -0.1238 | 0.1623 | 38.0337 | -0.7627 | 1.0000 |
| High.30 - ControlT.32 | 0.0898 | 0.1581 | 43.8936 | 0.5683 | 1.0000 |
| High.30 - Control.32 | -0.0960 | 0.1755 | 39.7100 | -0.5466 | 1.0000 |
| High.30 - Low.32 | -0.4548 | 0.1691 | 43.9963 | -2.6903 | 0.4598 |
| High.30 - Medium.32 | -0.2499 | 0.1691 | 43.9963 | -1.4783 | 0.9929 |
| High.30 - High.32 | -0.1128 | 0.1691 | 43.9963 | -0.6674 | 1.0000 |
| ControlT.32 - Control.32 | -0.1858 | 0.1581 | 43.8936 | -1.1754 | 0.9996 |
| ControlT.32 - Low.32 | -0.5446 | 0.1508 | 34.1036 | -3.6108 | 0.0836 |
| ControlT.32 - Medium.32 | -0.3397 | 0.1508 | 34.1036 | -2.2524 | 0.7511 |
| ControlT.32 - High.32 | -0.2027 | 0.1508 | 34.1036 | -1.3436 | 0.9973 |
| Control.32 - Low.32 | -0.3589 | 0.1691 | 43.9963 | -2.1227 | 0.8278 |
| Control.32 - Medium.32 | -0.1540 | 0.1691 | 43.9963 | -0.9107 | 1.0000 |
| Control.32 - High.32 | -0.0169 | 0.1691 | 43.9963 | -0.0998 | 1.0000 |
| Low.32 - Medium.32 | 0.2049 | 0.1623 | 38.0337 | 1.2623 | 0.9988 |
| Low.32 - High.32 | 0.3420 | 0.1623 | 38.0337 | 2.1068 | 0.8336 |
| Medium.32 - High.32 | 0.1371 | 0.1623 | 38.0337 | 0.8445 | 1.0000 |

**S4. Complementary graphs: *P. flava* fragment changes in surface area as a function of time**

**S4.1.** Changes in *P. flava* fragments surface area as a function of time for the first experimental stage, including Individual DOC additions as treatment.

**S4.2.** Changes in *P. flava* fragments surface area as a function of time for the second experimental stage, including simultaneous warming and DOC addition as treatments.
