# Supplementary figures and images for: Individual and combined effect of organic eutrophication (DOC) and ocean warming on the ecophysiology of the Octocoral Pinnigorgia flava
Source: PeerJ. 2023 Feb 17;11:e14812. doi: 10.7717/peerj.14812 (PMC9940650; doi:10.7717/peerj.14812)

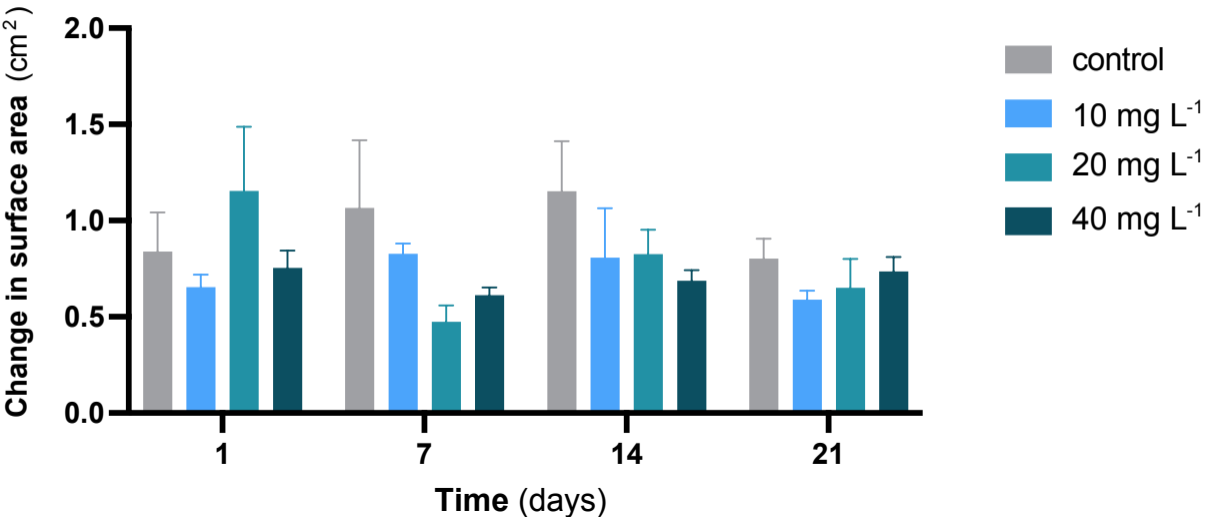

Supplement: Supplemental Information 1 — The raw data shows the ecophysiological response of the gorgonian Pinnigorgia flava to the effect of organic enrichment and increased water temperatures. In particular, the files report the measurements of oxygen concentration and the coral surfaces under the individual and the combined effect of DOC and temperatures throughout the whole experiment duration. [file peerj-11-14812-s001.zip › DOCTE - New Supplementary Materials/New - Supp - DOCTE - Surface_Area_Changes-DOC.pdf]

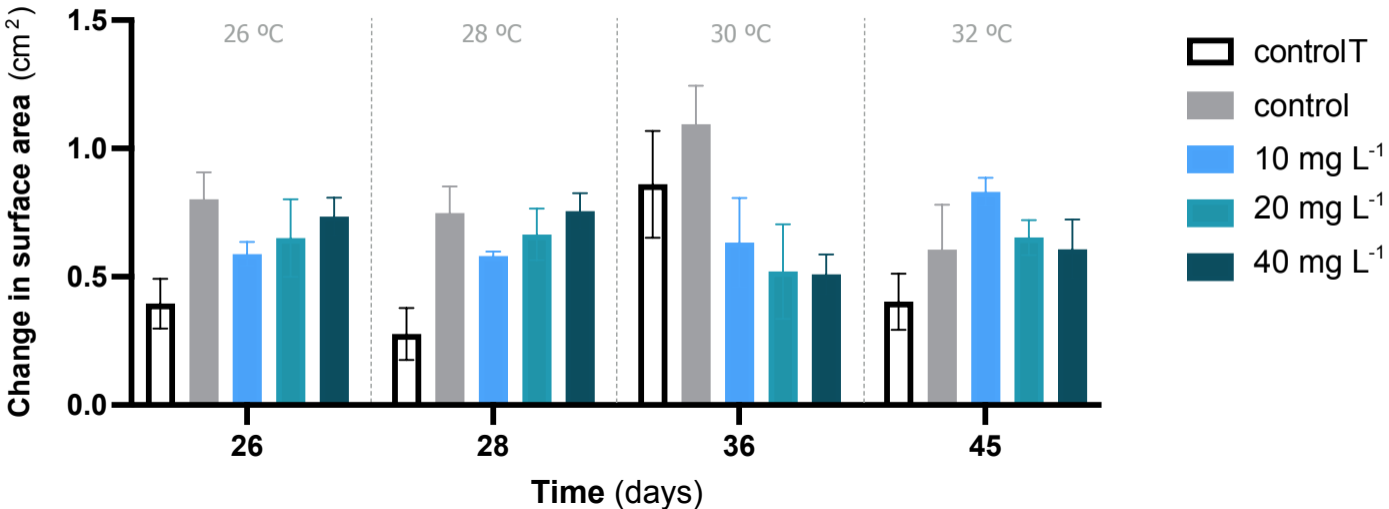

Supplement: Supplemental Information 1 — The raw data shows the ecophysiological response of the gorgonian Pinnigorgia flava to the effect of organic enrichment and increased water temperatures. In particular, the files report the measurements of oxygen concentration and the coral surfaces under the individual and the combined effect of DOC and temperatures throughout the whole experiment duration. [file peerj-11-14812-s001.zip › DOCTE - New Supplementary Materials/New - Supp - DOCTE - Surface_Area_Changes-DOCT.pdf]

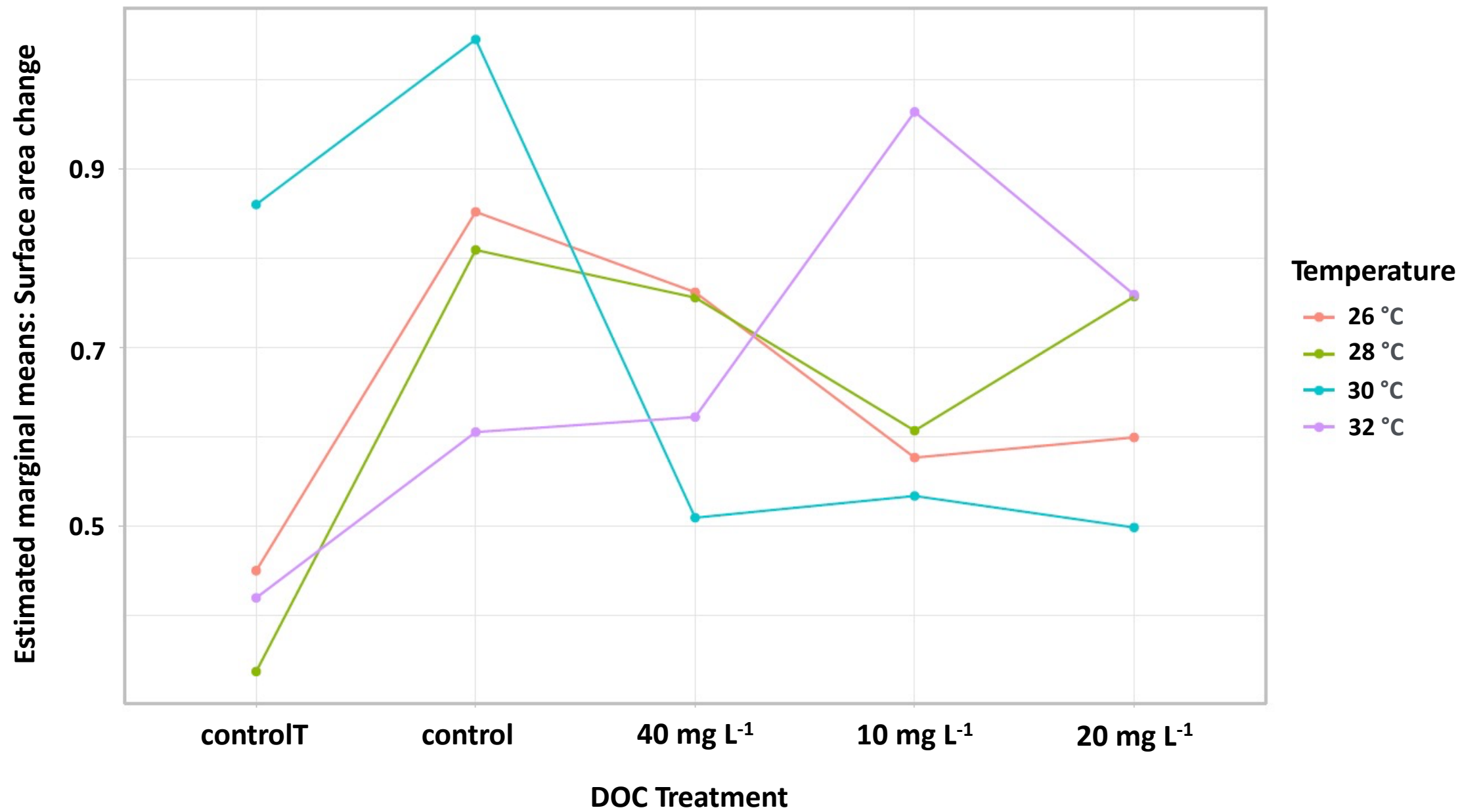

Supplement: Supplemental Information 1 — The raw data shows the ecophysiological response of the gorgonian Pinnigorgia flava to the effect of organic enrichment and increased water temperatures. In particular, the files report the measurements of oxygen concentration and the coral surfaces under the individual and the combined effect of DOC and temperatures throughout the whole experiment duration. [file peerj-11-14812-s001.zip › DOCTE - New Supplementary Materials/New - Supp - DOCTE - Marginal_means - Surface_Area_Changes-DOCT.pdf]
